# New free radical-initiated peptide sequencing (FRIPS) mass spectrometry reagent with high conjugation efficiency enabling single-step peptide sequencing

**DOI:** 10.1038/s41598-022-13624-0

**Published:** 2022-06-09

**Authors:** Sang Tak Lee, Hyemi Park, Inae Jang, Choong Sik Lee, Bongjin Moon, Han Bin Oh

**Affiliations:** 1 Department of Chemistry, Sogang University, Seoul, 04107 Korea; 2 Department of Toxicology and Chemistry, Scientific Investigation Laboratory, Criminal Investigation Command, Ministry of National Defense, Seoul, 04351 Korea

**Keywords:** Mass spectrometry, Proteomics

## Abstract

A newly designed TEMPO-FRIPS reagent, 4-(2,2,6,6-tetramethylpiperidine-1-oxyl) methyl benzyl succinic acid *N*-hydroxysuccinimide ester or *p-*TEMPO–Bn–Sc–NHS, was synthesized to achieve single-step free radical-initiated peptide sequencing mass spectrometry (FRIPS MS) for a number of model peptides, including phosphopeptides. The *p-*TEMPO–Bn–Sc–NHS reagent was conjugated to target peptides, and the resulting peptides were subjected to collisional activation. The peptide backbone dissociation behaviors of the MS/MS and MS^3^ experiments were monitored in positive ion mode. Fragment ions were observed even at the single-step thermal activation of the *p-*TEMPO–Bn–Sc–peptides, showing mainly **a**-/**x**- and **c**-/**z**-type fragments and neutral loss ions. This confirms that radical-driven peptide backbone dissociations occurred with the *p-*TEMPO–Bn–Sc–peptides. Compared to the previous version of the TEMPO reagent, *i.e.*, *o*-TEMPO*–*Bz–C(O)–NHS, the newly designed *p-*TEMPO–Bn–Sc–NHS has better conjugation efficiency for the target peptides owing to its improved structural flexibility and solubility in the experimental reagents. An energetic interpretation using the survival fraction as a function of applied normalized collision energy (NCE) ascertained the difference in the thermal activation between *p-*TEMPO–Bn–Sc– and *o*-TEMPO–Bz–C(O)– radical initiators. This study clearly demonstrates that the application of the *p*-TEMPO–Bn–Sc– radical initiator can improve the duty cycle, and this FRIPS MS approach has the potential to be implemented in proteomics studies, including phosphoproteomics.

## Introduction

A variety of electron-based and radical-based dissociation tandem mass spectrometry methods have been developed and found to be very useful for characterizing the post-translational modifications (PTMs) of peptides/proteins and for top-down/middle-down/bottom-up protein analysis, the structural analysis of proteins and glycan analysis^1–8^. Compared with electron-based tandem mass spectrometry methods, such as electron capture (transfer) dissociations (ECD/ETD, or collectively ExDs), radical-based tandem mass spectrometry (RxD) methods have a clear advantage in that they generally do not require additional instrumentation. For RxD methods, many different strategies have been used. For example, UV-photodissociation of a radical precursor, photodetachment of an electron in anionic species, and collisional activation (CA) of labile radical precursors are representative methods^9–12^.

Among these methods, our group has paid attention to the method listed last, in which a radical site is introduced by the homolytic cleavage of a labile bond by CA in a peptide with a radical precursor. This method was originally developed independently by two research groups, *i.e.*, the Porter and Beachamp groups, and denoted as free radical-promoted peptide sequencing and free radical-initiated peptide sequence (FRIPS), respectively ^13–15^. In particular, an azo group and a 2,2,6,6-tetramethylpiperidine-1-oxyl (hereafter denoted as TEMPO) group have been the two most widely used FRIPS precursor moieties^13,16–18^. The applicability of these moieties to the mass spectrometric analysis of peptides and the dissociative mechanism of radical precursor-conjugated peptides have been extensively investigated^19–21^.

Our group introduced *ortho*-[(2,2,6,6-tetramethylpyperidine-1-oxyl) methyl]benzoic acid *N*-hydroxysuccinimide ester (*o*-TEMPO–Bz–C(O)–NHS) as a FRIPS reagent^22^. The TEMPO moiety, which is known to have a high radical stability, plays an important thermodynamic role in inducing the homolytic cleavage of the C–O bond between the benzylic carbon and TEMPO oxygen, leading to the exclusive formation of (TEMPO• and) •Bz–C(O)–peptide species. Once radical sites are introduced into the peptides in the form of •Bz–C(O)–peptide species, radical reactions readily ensue upon the subsequent CA to result in extensive peptide backbone dissociations or side-chain fragmentations. Generally, an initial benzyl radical site migrates to the other position of the peptide backbone or side chain through a hydrogen abstraction reaction, which eventually leads to the β-cleavage of the C–C_α_, N–C_α_, C_α_–C_β_ and C_γ_–C_δ_ bonds^20,23,24^. Such cleavages at these bonds result in the production of ***a***-/***x***- and ***c***-/***z***-type fragments, side-chain loss fragments, or neutral loss fragments^21,24–26^.

This so-called TEMPO-assisted FRIPS MS approach has shown some interesting characteristics as a tandem mass spectrometry tool. First, with this approach, disulfide bonds were shown to be exclusively cleaved prior to peptide backbone fragmentations^15,27^. This feature is expected to be useful for the disulfide bond mapping of antibodies. Second, like the other ExDs and RxD methods, TEMPO-assisted FRIPS MS preserves phosphate groups in the analysis of phosphopeptides while fragmenting peptide backbones^28^. Thus, this approach has great potential for use in phosphoproteomic research. Third, due to its excellent peptide backbone fragmentation capability, a TEMPO-based FRIPS reagent was adopted in the design of a protein cross-linker that is usually used for revealing the rough 3D structure of proteins^20,29–32^. Last, the improvement of FRIPS MS radical-driven tandem mass spectrometry performance could be achieved with the introduction of a new constituent in the molecular design. For example, Gasper et al. incorporated a pyridine ring between the TEMPO radical precursor and the N-hydroxysuccinimide so that the proton mainly resided on the pyridine ring, thus limiting the charge-driven fragmentations and enabling single-step FRIPS MS: the so-called charge localized FRIPS (CL-FRIPS)^33^.

With these useful features of TEMPO-assisted FRIPS MS, this approach needs to be improved to be used more widely in peptide/protein MS research. One of the drawbacks of TEMPO-assisted FRIPS MS is the lower duty cycle that requires two CA steps to observe peptide backbone fragments in positive-ion low collision-energy experiments; the first CA step, the generation of a radical site; and the second CA step, the radical-driven peptide backbone dissociations. Although a single-step positive-ion FRIPS MS could be achieved through higher collision-energy experiments, *e.g*., MS/MS experiments of a hybrid quadrupole time-of-flight (Q-TOF) mass spectrometer and higher collision-energy dissociations (HCDs) of an orbitrap instrument, this issue needs to be addressed^34^. A key clue to resolve this issue could be found in the previous negative-ion FRIPS MS method using *o*-TEMPO–Bz–C(O)–NHS. Due to the reversed energy ordering between the MS/MS and MS^3^ steps, a single-step FRIPS MS induced extensive peptide backbone dissociations in negative-ion mode^35^. In negative-ion mode, the MS/MS step required a higher collision energy than in positive-ion mode. In other words, in negative-ion mode, the TEMPO• and •Bz–C(O)–peptide species formed in the MS/MS step were destabilized with respect to those in positive-ion mode. Based on this rationale, a factor to destabilize the •Bz–C(O)–peptide species was considered, *i.e.*, the introduction of a succinate group between the benzene ring and NHS conjugation linker, instead of the carbonyl group that can stabilize the radical character at the ortho benzylic position through a resonance effect. Furthermore, the introduction of a succinate group can improve the solubility of the conjugated peptides with the addition of basic oxygen atoms. With these ideas, we propose an improved radical-precursor reagent in the present paper, which is 4-(2,2,6,6-tetramethylpiperidine-1-oxyl) methyl benzyl succinic acid N-hydroxysuccinimide (*para*-TEMPO–benzyl–succinate–NHS or *p-*TEMPO–Bn–Sc–NHS).

**Figure 1:**
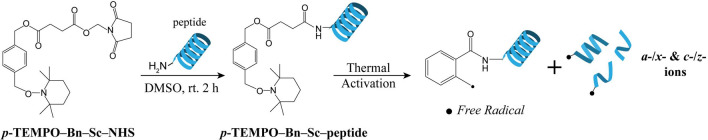
Single-step FRIPS MS of *p*-TEMPO–Bn–Sc–peptide.

Herein, we will explore and discuss the peptide dissociation behavior of the newly suggested *p-*TEMPO–Bn–Sc–peptides and their conjugation efficiency. In particular, the feasibility of the single-step peptide sequencing ability of the *p-*TEMPO–Bn–Sc–peptides (Fig. 1) will be examined in detail, and the applicability of this method in investigating phosphorylated peptides will be explored.

## Experimental details

### Materials

Four model peptides, *i.e.*, angiotensin II (DRVYIHPF), kinetensin (IARRHPYFL), glycoprotein IIb fragment (296–306) (TDVNGDGRHDL), and des-Pro^2^-bradykinin (RPPGFSPFR), with purity higher than 99% were purchased from Bachem AG (Bubendorf, Switzerland) and were used without further purification. LC–MS grade organic solvents (water, methanol, acetonitrile) were purchased from Fisher Scientific (Hampton, NH, USA). Anhydrous dimethyl sulfoxide, triethylammonium bicarbonate buffer (1 M), 99.5% formic acid, dithiothreitol, iodoacetamide, Tris–HCl buffer, 8 M urea solution, α-casein, and chicken egg albumin were purchased from Sigma Aldrich (St. Louis, MO, USA). Mass spectrometry grade Trypsin-ultra™ was purchased from New England Biolabs (Ipswich, MA, USA). Peptide desalting and phosphopeptide enrichment were carried out using a HyperSep™ C18 SPE cartridge and High-Select™ TiO_2_ phosphopeptide enrichment kit from Thermo Scientific (San Jose, CA, USA), respectively. Fused silica capillary tubing with an outer diameter of 360 µm and an inner diameter of 100 µm was purchased from Polymicro Technologies (Phoenix, AZ, USA). Microcross, microtee and microtight units for connecting the capillary tubing were purchased from IDEX Health and Science (Oak Harbor, WA, USA).

### Synthesis of *p*-TEMPO–Bn–Sc–NHS

A new radical initiator, 4-(2,2,6,6-tetramethylpiperidine-1-oxyl) methyl benzyl succinic acid *N*-hydroxysuccinimide ester (*para*-TEMPO–Bn–Sc–NHS or *p*-TEMPO–Bn–Sc–NHS), was synthesized as follows (see Supplementary Fig. 1). Unless otherwise stated, all the chemicals used in each preparation step described below were purchased from Sigma–Aldrich (St. Louis, MO, USA).

Preparation of **2**: HCl_conc_ (5.00 equiv.) was added at room temperature to a stirred suspension of 1,4-benzenedimethanol (1.00 equiv.) in toluene. The resulting solution was stirred for 12 h at room temperature. Then, the solution was washed with aqueous NaHCO_3_ and water, the organic phase was separated, and the aqueous layer was extracted with CH_2_Cl_2_. The combined organic layers were dried over anhydrous Na_2_SO_4_ and concentrated *in vacuo* to provide the desired product, (4-chloromethylphenyl)methanol.

Preparation of **3**: To a Schlenk flask was added (4-chloromethylphenyl)methanol (3.40 mmol), TEMPO (4.08 mmol), Cu (OTf)_2_ (0.033 mmol), copper powder (3.40 mmol), 2,2-bipyridine (1.36 mmol), and benzene. The reaction mixture was degassed by bubbling argon for 10 min and heated at 70 ℃ for 16 h. After cooling the reaction mixture to room temperature, it was filtered through a short pad of silica gel using EtOAc. The filtrate was concentrated *in vacuo*, and the residue was purified by flash column chromatography using hexane:EtOAc (2:1) to give the product as a white solid.

Preparation of **4**: Under argon atmostphere, Compound **3** (1.00 equiv.) and NaBH_4_ (5.00 equiv.) were placed in a 100 mL round bottom flask equipped with a stirring bar. Ethanol was then added to the flask at room temperature. The mixture was stirred for 2 h at room temperature. The reaction was quenched by adding 10% HCl at room temperature. The solution was then extracted with CH_2_Cl_2_. The collected organic layers were dried over MgSO_4_ and concentrated using a rotary evaporator. The crude product was purified by flash column chromatography using hexane:EtOAc (2:1) to give the product.

Preparation of **5**: In a screw-capped culture tube, Compound **4** (1.00 equiv.), succinic anhydride (1.00 equiv.), and 4-dimethylaminopyridine (0.5 equiv.) were placed and diluted with benzene under a N_2_ atmosphere. The mixture was heated for 5 h under reflux. The solution was then extracted with CH_2_Cl_2_. The collected organic layers were dried over MgSO_4_ and concentrated using a rotary evaporator to give the desired product **5**.

Preparation of **6**: Compound **5** (1.16 mM), *N*-hydroxysuccinimide (1.16 mmol), and DMAP-TsOH (0.058 mmol) were dissolved in dry CH_2_Cl_2_ under a nitrogen atmosphere. A solution of *N,N’*-dicyclohexylcarbodiimide (DCC) (1.39 mmol) was slowly added to the solution at 0 °C. After 12 h of stirring, the reaction mixture was filtered, and the filtrate was concentrated to give a crude product. Purification of the crude product by column chromatography on silica gel (Hex:EtOAc = 1:1) gave the product as a white solid.

### Protein digestion and enrichment of phosphopeptides

Phosphopeptides from two different proteins, chicken egg albumin and alpha-casein, were enriched using the following procedure. One milligram of each protein was dissolved in a denaturing solution containing 100 mM Tris–HCl and 8 M urea. The disulfide bonds of the chicken egg albumin were reduced by adding 5 mM dithiothreitol (DTT) and incubating the solution for 1 h at 56 °C. The sample was cooled, and the reduced thiol groups were further alkylated with 14 mM iodoacetamide (IAA) in the dark for 30 min. Excess IAA was removed by adding 5 mM DTT to the solution, and then each protein solution was diluted to a final concentration of 1 M urea with 25 mM Tris–HCl. Then, each protein solution was digested using sequencing-grade trypsin (1/50 of target protein amount) and incubated for 18 h at 37 °C. The digestion was quenched by adding 0.1% formic acid to the solution, and each solution was centrifuged at 2,500 rpm for 10 min to form a cloudy pellet below the supernatant, which was then discarded. The resulting peptides were desalted using a 1 mL HyperSep™ C18 SPE cartridge (Thermo Scientific, San Jose, CA, USA) and then dried in a SpeedVac for the next phosphopeptide enrichment. Phosphopeptide enrichment was carried out using a High-Select™ TiO_2_ phosphopeptide enrichment kit from Thermo Scientific, and a detailed procedure can be found in the manufacturer’s protocol. The enriched phosphopeptides were then dried in a SpeedVac overnight and resuspended in 50 μL of water for the conjugation of *p-TEMPO–Bn*–Sc–NHS.

### Peptide conjugated with *p*-TEMPO–Bn–Sc–NHS

Model peptides and enriched phosphopeptides were dissolved in HPLC grade water to a concentration of 1 μg/μL for use as stock solutions. A stock solution of *p*-TEMPO–Bn–Sc–NHS was prepared in anhydrous DMSO at a concentration of 5 µg/μL. A total of 1 µg (= 1 μL) of a model peptide was mixed with 1 μL of 0.1 M triethylammonium bicarbonate buffer solution (~ pH 8.5) and combined with 18 µL of *p*-TEMPO–Bn–Sc–NHS solution. The water content of the whole solution was kept below 10% to prevent the hydrolysis of *p*-TEMPO–Bn–Sc–NHS and to enable rapid and efficient conjugation of the radical initiator to the peptides. The mixture was allowed to stand for an appropriate period of time. In the experiment carried out to test the conjugation efficiency of *p*-TEMPO–Bn–Sc–NHS to a model protein, the mixture was allowed to react for several different periods of time, from 0.5 h to 30 h. A 5 µL aliquot of the reacted mixture was dried under vacuum using a SpeedVac and resuspended in 100 µL of solution composed of water and methanol (50:50 v/v) with 0.1% formic acid.

### Mass spectrometry and LC–MS/MS

TEMPO-assisted FRIPS MS for the model peptides was performed with a linear ion trap mass spectrometer (LTQ XL, Thermo Scientific, San Jose, CA, USA; Advanced Bio-Interface Core Research Facility, Sogang University) in positive ion mode. In analyzing *p*-TEMPO–Bn–Sc–NHS conjugated peptides, a prepared sample was directly infused through a nanoelectrospray ionization (nano-ESI) source at a flow rate of 500 nL/min using a built-in syringe pump. A nanospray emitter was prepared by pulling one end of a fused silica capillary (100 µm i.d., 360 µm o.d.) during flame heating to make a sharp needle and by cutting the needle end to have a tip thickness of less than 10 µm. A precursor ion was isolated and then subjected to further MS/MS and MS^3^ by collision-activation dissociation (CAD) using MS parameters as follows: nanoESI voltage, + 2.0 kV; capillary temperature, 200 °C; tube lens offset voltage, + 250 V; isolation width, 2–3 Da; capillary voltage, + (45–50) V; activation Q, 0.25; activation time, 30 ms. The ESI mass spectra were obtained by averaging 20–30 scanned spectra.

For the collisional activation of the *p-TEMPO–Bn*–Sc–conjugated phosphopeptide enriched from chicken egg albumin and alpha-casein, an Exploris 240 orbitrap mass spectrometer (Thermo, San Jose, CA, USA) was utilized with the following parameters: an ion spray voltage of + 3.8 kV; an ion transfer tube temperature of 320 °C; a flow rate of 5 μL/min; an RF lens of 120%; and a resolving power of 120,000. Tandem mass spectrometry (MS/MS) was performed using the following parameters: an isolation width of 1 m/z, a max inject time of 200 ms, and normalized collision energy (NCE) of 25 (a.u.). The spectra were averaged 500 to 1000 scans. Tandem mass spectrometry (MS/MS) was performed using the following parameters: an isolation width of 1 m/z, a max injection time of 200 ms, and a normalized collision energy (NCE) of 25 (a.u.). The spectra were averaged over 500 to 1000 scans. Tandem mass spectrometry (MS/MS) data were analyzed using MASH Explorer developed by the Ge group^36^. Spectral deconvolution and fragment ion searches were carried out with enhanced-THRASH (eTHRASH) with the following parameters: an S/N ratio of 3; a fragment ion tolerance of 0, ± 1, ± 2 Da; a fit score of 60%; and a 15 ppm error cutoff.

For the comparison of FRIPS MS of *p-TEMPO–Bn*–Sc–conjugated phosphopeptides with electron-transfer dissociation (ETD) MS, electron-transfer/higher-energy collision dissociation (EThcD) fragmentation was carried out for the phosphopeptides using a separate instrument. A nano-flow ultra-high-performance liquid chromatography (UHPLC) system (UltiMate 3000 RSLCnano, Thermo Fisher Scientific) in combination with the mass spectrometer (Orbitrap Eclipse™ Tribrid™, Thermo Fisher Scientific: Korea Basic Science Institute in Ochang, Korea) was used for EThcD analyses of the phosphopeptides. Phosphopeptides were injected and separated on EASY-Spray PepMap™ RSLC C18 Column (2 µm, 100 Å, 75 µm × 50 cm, Thermo Fisher Scientific) operated at 50 °C. A gradient from 5.0 to 95% mobile phase B over 110 min with a flow rate of 250 nL/min was applied with mobile phases A: H_2_O/FA (99.9:0.1, v:v) and B: ACN/H_2_O/FA (80:19.9:0.1, v:v:v). ESI voltage was 1850 V, and the ion transfer tube temperature was 275 °C.

UHPLC-MS/MS data were acquired using a data dependent top-speed mode comprising of a full scan followed to maximize the number of MS^2^ scans during 3 s of cycle time. Full scan was detected by the orbitrap analyzer at a resolution of 120,000 with the mass range of *m/z* 350–2000. An automatic gain control (AGC) target value was 4e^5^, and the maximum injection time was 100 ms. EThcD MS/MS spectra were obtained by the orbitrap analyzer with the following parameters: resolution of 30,000; isolation window of 2 m*/z*; reaction time of 100 ms; supplemental activation collision energy of 15%; maximum injection time of 250 ms; and ETD reagent target value of 2.0e^5^.

## Results and discussion

### Conjugation efficiency

In peptide MS applications involving a conjugation reagent, the efficiency of coupling a reagent with peptides often plays an important role in determining the overall efficiencies of the peptide/protein identifications and characterizations. Thus, we optimized the solvent composition and reaction time to improve the conjugation efficiencies. Figure 1 shows the conjugation efficiencies as a function of reaction time, which were monitored using three different solvent compositions: (a) water:DMSO (v/v 10:90), (b) water:DMSO (v/v 50:50), and (c) water:DMSO (v/v 90:10). In this evaluation, both radical initiator reagents, *i.e.*, *o*-TEMPO–Bz–C(O)–NHS and *p*-TEMPO–Bn–Sc–NHS, were conjugated to kinetensin (IARRHPYFL), and the conjugation efficiency was monitored at 12 different time points from 0 to 30 h. The conjugation efficiency was calculated using the following equation.

1 $${\text{Conjugation}}\;{\text{Efficiency (\% ) = }}\frac{Aconj.,( + 1) + Aconj.,( + 2)}{{Anon - conj.,( + 1) + Anon - conj.,( + 2) + Aconj.,( + 1) + Aconj.,( + 2)}} \times 100$$

*A*_*conj*_.,(+ 1):mass peak abundance of singly protonated TEMPO conjugated peptide; *A*_*conj.*_,(+ 2):mass peak abundance of doubly protonated TEMPO conjugated peptide; *A*_*non—conj*_.,(+ 1):mass peak abundance of singly protonated peptide without conjugation ; *A*_*non—conj*_.,(+ 2):mass peak abundance of doubly protonated peptide without conjugation where the sum of the mass peak abundances of singly and doubly protonated peptides in the conjugated form were divided by the sum of those in both conjugated and nonconjugated forms.

**Figure 2:**
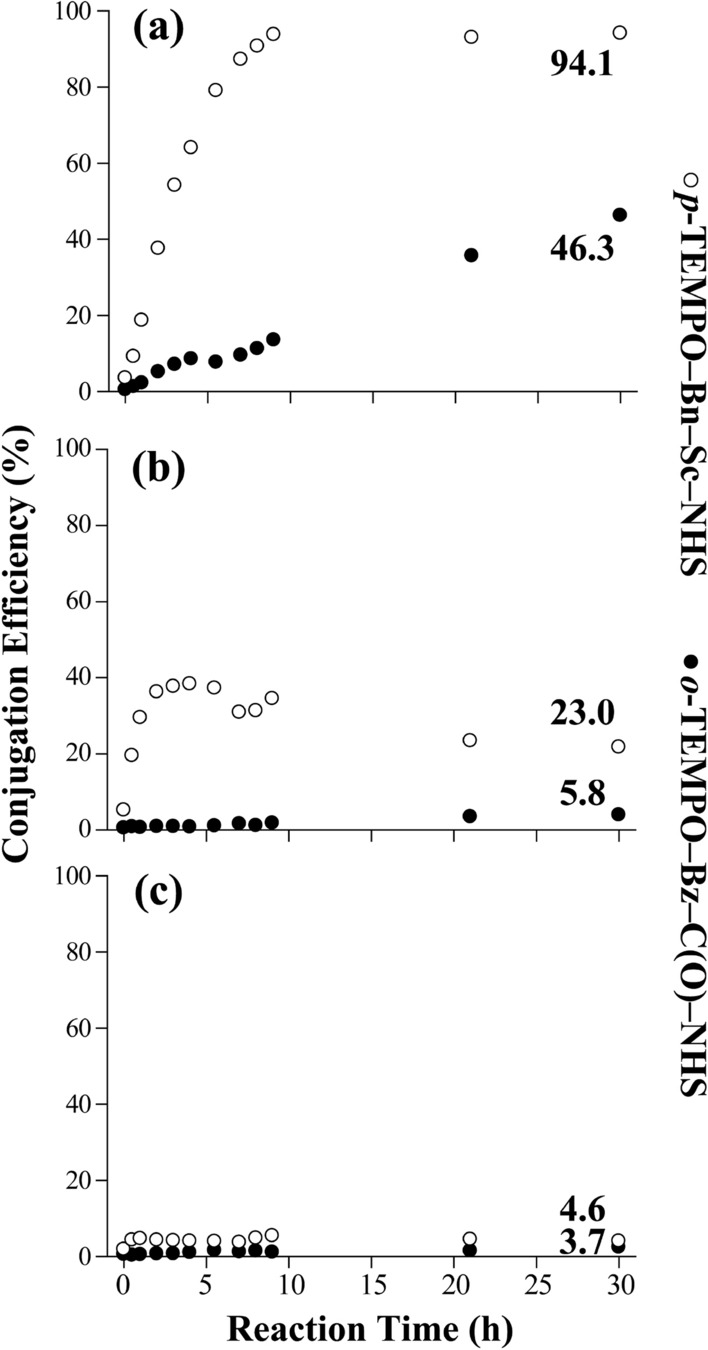
Conjugation efficiencies of *p*-TEMPO–Bn–Sc–NHS (empty circles) and *o*-TEMPO–Bz–C(O)–NHS (filled circles) to kinetensin (IARRHPYFL) under three different reaction conditions: (**a**) water:DMSO (v/v 10:90), (**b**) water:DMSO (v/v 50:50), and (**c**) water:DMSO (v/v 90:10).

*p-*TEMPO–Bn–Sc–NHS showed a very high conjugation efficiency (95%) after 10 h under the condition where the water content of the reaction media was kept at 10%, whereas *o*-TEMPO–Bz–C(O)–NHS reached only 40% conjugation efficiency even after 30 h under the same conditions (see Fig. 2a). When the water content increased to 50%, the conjugation efficiency of *p-*TEMPO–Bn–Sc–NHS dropped to approximately 40% (max) after 5 h of reaction. In the case of *o*-TEMPO–Bz–C(O)–NHS, the efficiency dropped even more, showing only 5.8% even after 30 h of reaction (Fig. 2b). With a water content of 90%, both radical initiator reagents showed very poor conjugation efficiencies of less than 5% (Fig. 2c). The lower conjugation efficiency with the higher water content in the reaction media can be attributed either to the competitive hydrolysis of the reagent by water or the decreased solubility of the reagents. However, it is necessary to use some amount of water to fully dissolve peptides, and thus a solution with 90% DMSO and 10% water was used as a solvent in the following experiments.

*p*-TEMPO–Bn–Sc–NHS exhibits a higher conjugation efficiency because it has an aliphatic NHS ester group with a long succinate (Sc) linker, which is more reactive than the aromatic benzoyl NHS ester group in *o-*TEMPO–Bz–C(O)–NHS. In addition, the slower conjugation kinetics of *o-*TEMPO–Bz–C(O)–NHS can be partially ascribed to the more sterically hindered *ortho-*arrangement of the substituents in which the benzoyl (Bz) NHS group is close to the *ortho*-TEMPO–methyl group. The introduction of the succinyl (Sc) linker in *p-*TEMPO–Bn–Sc–NHS also seemed to enhance its solubility in the DMSO:H_2_O mixed solvent system. To summarize, the newly developed *p*-TEMPO–Bn–Sc–NHS radical initiator reagent has exhibited faster and more effective conjugation to the *N*-termini of peptides than the previous *o*-TEMPO–Bz–C(O)–NHS radical initiator reagent.

### Peptide backbone dissociations as a function of CA energy

**Figure 3:**
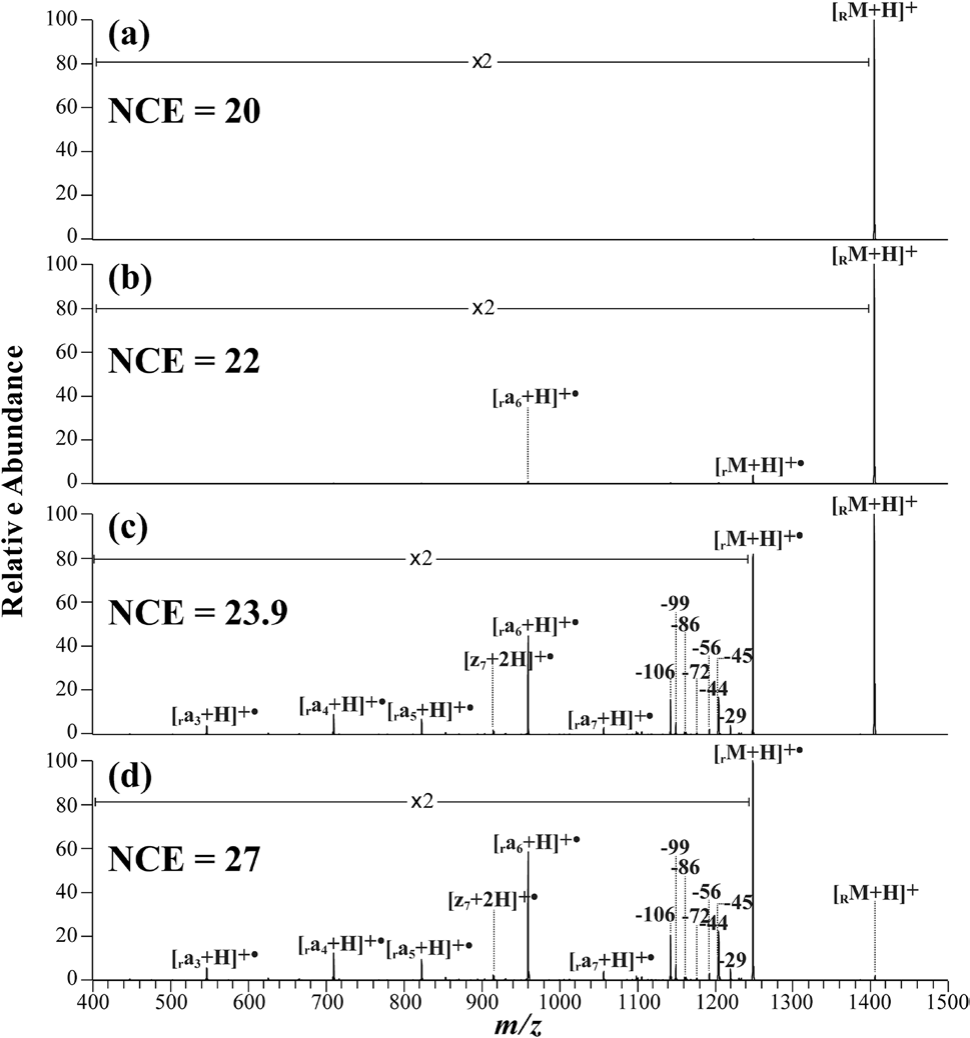
MS/MS spectra of singly protonated *p*-TEMPO–Bn–Sc–angiotensin II (DRVYIHPF) at different normalized collision energies (NCEs, a.u.): (**a**) 20, (**b**) 22, (**c**) 23.9 and (**d**) 27. In the mass spectra, the subscript ‘R’ indicates that the *p-*TEMPO–Bn–Sc– group was conjugated to the N-terminal primary amine of peptides, and the subscript ‘r’, *e.g*., [_r_M + H]^+^, denotes the •Bn–Sc– group, which was formed by the homolytic cleavage of the C–O bond between the benzyl carbon and the oxygen of the TEMPO group.

To better understand what happened in the one-step TEMPO-assisted FRIPS process, it is necessary to follow the dissociation behavior as a function of the provided collision energy (CA). Figure 3 shows the FRIPS MS/MS spectra of singly protonated *p*-TEMPO–Bn–Sc–angiotensin II (DRVYIHPF) at four different normalized collision energies (NCEs): (a) 20, (b) 22, (c) 23.9, and (d) 27. Here, the subscript ‘R’ indicates that the *p*-TEMPO–Bn–Sc–NHS group was conjugated to the N-terminal primary amine of the peptide, and ‘r’ indicates the •Bn–Sc–peptide with free radicals formed by the homolytic cleavage of the C–O bond between the benzyl carbon and the oxygen. To facilitate a comparison among these spectra, the spectra are presented with the same degree of magnification in the *m/z* range of 400 to 1400. At an NCE of 20, no fragmentation occurred. When the NCE was increased to 22, *p*-TEMPO–Bn–Sc–angiotensin II, *i.e.*, [_R_M + H]^+^, underwent homolytic cleavage, leading to the formation of the •Bn–Sc–angiotensin II radical precursor ([_r_M + H]^+•^) at *m/z* 1249.6 and producing the peptide backbone fragment of [_r_a_6_ + H]^+•^ at *m/z* 960.7. In a separate CA experiment on [_r_M + H]^+•^, [_r_***a***_6_ + H]^+•^ was found to be the first product ion arising from the fragmentation of [_r_M + H]^+•^. Thus, the observation of [_r_***a***_6_ + H]^+•^ in Fig. 3b at NCE 22 indicates that the thermal energy provided at NCE 22 was sufficient to lead to the generation of •Bn-Sc-angiotensin II and to result in consecutive peptide fragmentation to produce [_r_***a***_6_ + H]^+•^. Figure 3c and d are the MS/MS spectra obtained at NCEs of 23.9 and 27, respectively. NCE 23.9 corresponds to NCE_1/2,_ at which half of the precursor ion was fragmented into product ions and its neutral/radical loss peaks (see below). As the NCE increased further, a series of ***a***-ions and radical/neutral loss peaks, for example, [_r_***a***_3_ + H]^+•^, [_r_***a***_4_ + H]^+•^, [_r_***a***_5_ + H]^+•^, [_r_***a***_6_ + H]^+•^, [_r_***a***_7_ + H]^+•^, –29, –44, –45, –56, –72, –86, –99 and –106 Da, arose, and their abundances increased in proportion to the collision energy applied.

### Dissociation energetics of TEMPO-conjugated peptides

**Figure 4:**
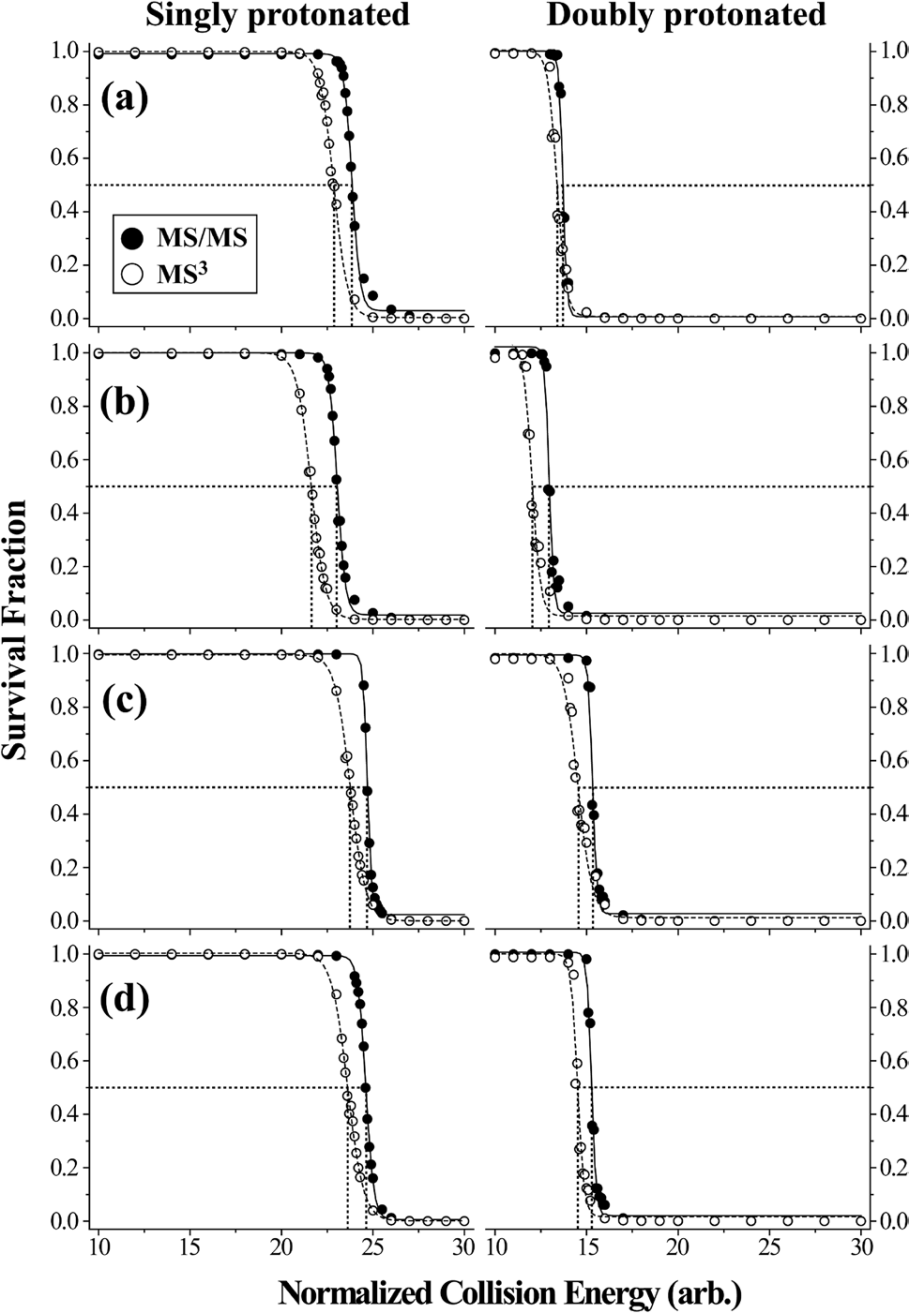
Survival fractions of *p*-TEMPO–Bn–Sc conjugated peptides: (**a**) angiotensin II, (**b**) des-Pro^2^-bradykinin, (**c**) glycoprotein IIb fragment (296–306) and (**d**) kinetensin. The scale on the left y-axis indicates the survival fractions of singly protonated peptides, while that on the right y-axis shows those of doubly protonated peptides.

Figure 4 shows the survival fraction curves of four different *p*-TEMPO–Bn–Sc–peptides: (a) angiotensin II, (b) des-Pro^2^-bradykinin (RPGFSPFR), (c) glycoprotein IIb fragment 296–306 (TDVNGDGRHDL) and (d) kinetensin (IARRHPYFL). The survival fraction is defined as the abundance of the precursor ion surviving at the NCE applied (see Eq. 2).

2 $${\text{Survival}}\;{\text{Fraction = }}\frac{{A{\text{precursor ion}}}}{{A{\text{precursor ion + }}\sum {A{\text{product ion}}} }}$$

where *A*_*precursor* ion_ and *A*_*product ion*_ refer to the abundances of the precursor and product ions, respectively. The precursor ions in the MS/MS and MS^3^ spectra are *p*-TEMPO–Bn–Sc–peptide and •Bn–Sc–peptide, respectively. The most notable feature in the survival fraction curves is that the NCEs required for MS/MS (or the first CA process) were generally higher than those required for MS^3^ (the second CA process) for both singly and doubly protonated conjugated peptide precursor ions. In the first CA step, the *p*-TEMPO• radical is detached from the *p*-TEMPO–Bn–Sc–peptide to generate the •Bn–Sc–peptide. On the other hand, in the second CA step, thermal energy was utilized for the dissociation of the peptide backbone. To easily compare the dissociation energetics of the first and second CA processes, NCE_1/2_ was obtained by sigmoidal curve fitting and is listed in Table 1. As readily noticed in this table, the NCE_1/2_ values in MS/MS were higher than those in MS^3^ for all the peptides.

**Table 1:** NCE_1/2_ values of *p*-TEMPO–Bn–Sc–peptides at their singly and doubly protonated states in MS/MS and MS^3^.

Peptide	NCE_1/2_					
	Singly protonated			Doubly protonated		
	MS/MS	MS^3^	Δ	MS/MS	MS^3^	Δ
Angiotensin II	23.9	22.9	1.0	13.7	13.4	0.3
Des-Pro2-bradykinin	23.0	21.6	1.4	13.0	12.0	1.0
Glycoprotein IIb fragment	24.7	23.8	0.9	15.3	14.5	0.8
Kinetensin	24.6	23.6	1.0	15.3	14.5	0.8

*All NCE_1/2_ values are displayed in arbitrary unit.

Interestingly, this energy ordering, in which the NCE_1/2_ required in the first CA was higher than that in the second CA step, was also observed in our previous negative-ion *o*-TEMPO–Bz–C(O)–FRIPS studies, wherein a single-step CA also produced extensive radical-driven peptide backbone dissociations. In contrast, in the previous positive-ion *o*-TEMPO–Bz–C(O)–FRIPS studies, wherein two-step CAs were necessary to observe peptide backbone dissociations in the low collision-energy ion-trap experiments, the energy ordering was different from the present case. These observations clearly indicate that the relative energy ordering between the first and second CAs plays a critical role in determining how many CA step(s) are required to induce peptide backbone fragmentations.

Then, the question arises of what causes this observed relative energy ordering reversal. It is thought that the *p*-TEMPO–Bn–Sc– FRIPS MS requires a higher CA than the *o*-TEMPO–Bz–C(O)– FRIPS MS. In the case of *o*-TEMPO–Bz–C(O)– FRIPS MS, the generated benzyl radical, *i.e.*, the •Bz–C(O)–peptide, can be stabilized by the carbonyl group through the resonance effect, and thus, less CA is required to generate the •Bz–C(O)–peptide. In contrast, due to the absence of the carbonyl group and the presence of the succinyl group, the benzyl radical generated in the •Bn–Sc–peptide cannot be stabilized, and thus, a higher CA should be required in the *p*-TEMPO–Bn–Sc– FRIPS MS. Once the •Bn–Sc–peptide is generated through the higher CAs, it contains extra thermal energy that is in enough excess to crossover the energy barriers, leading to extensive peptide backbone fragmentations. This is in contrast to the •Bz–C(O)–peptide, which does not have enough thermal energy to result in peptide backbone dissociations, requiring an extra CA step for the completion of the FRIPS MS.

### Low energy MS/MS and MS^3^

To characterize the peptide fragmentation behavior of *p*-TEMPO–Bn–Sc– conjugated peptides, low-energy MS/MS and MS^3^ experiments were carried out in positive-ion mode for a number of peptides (angiotensin II, des-Pro^2^-bradykinin, glycoprotein IIb fragment 296–306, and kinetensin) conjugated with *p*-TEMPO–Bn–Sc–NHS.

### Angiotensin II

**Figure 5:**
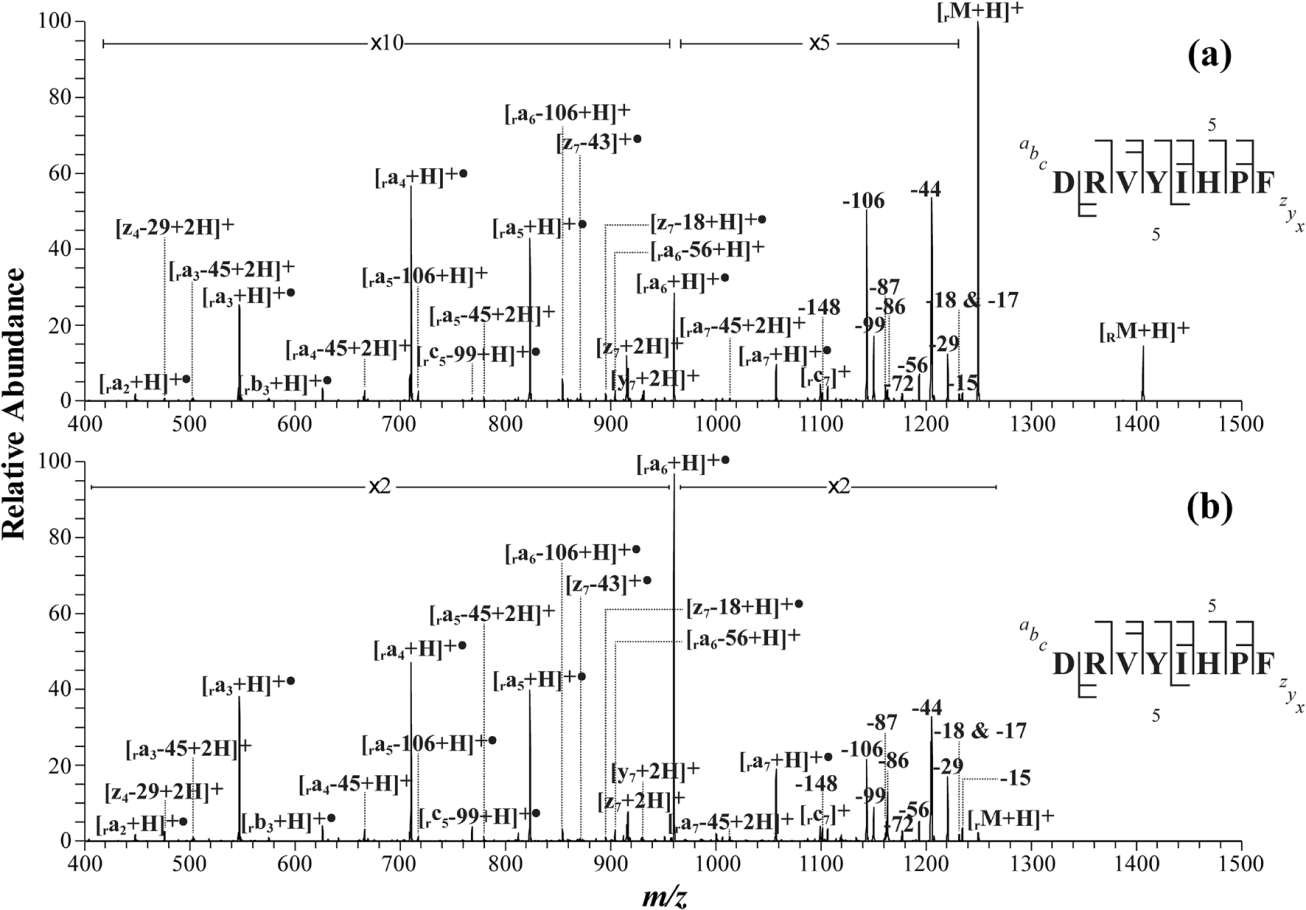
(**a**) MS/MS and (**b**) MS^3^ spectra of singly protonated *p*-TEMPO–Bn–Sc–angiotensin II (DRVYIHPF) at normalized collision energy of 25 (arbitrary units or a.u.).

Figure 5 shows (a) MS/MS and (b) MS^3^ spectra of singly protonated *p*-TEMPO–Bn–Sc–angiotensin II (DRVYIHPF, *m/z* 1405.8, [_R_M + H]^+^) acquired under *low collision-energy* conditions of the linear ion-trap instrument. In the MS/MS spectrum (Fig. 5a), it is noticeable that unlike *o*-TEMPO–Bz–C(O)–angiotensin II, which exclusively yielded •Bz–C(O)–angiotensin II radical ion, *i.e.*, [_r_M + H]^+•^, at the first collision activation (MS/MS) step, a single application of *low energy* collisional activations to *p*-TEMPO–Bn–Sc–angiotensin II produced a large number of peptide backbone fragments and neutral loss ions as well as [_r_M + H]^+•^ at *m/z* 1249.6 (*i.e.*, •Bn–Sc–angiotensin II). This result is in contrast to the case of *o*-TEMPO–Bz–C(O)–DRVYIHPF, for which two-step collisional activations were required to observe extensive peptide backbone fragments and neutral loss ions. Extensive single-step peptide backbone fragmentations have never been observed previously with low collision-energy MS/MS in positive ion mode, although higher-energy collision MS/MS or negative ion MS/MS were previously shown to induce extensive peptide backbone dissociations in a single step.

Another important observation is that the MS/MS spectrum of singly protonated *p*-TEMPO–Bn–Sc–angiotensin II is almost identical to its MS^3^ spectrum (*i.e.*, tandem mass spectrum of [_r_M + H]^+•^, Fig. 5b) in terms of the various fragments and their relative abundances, except for a few fragments, such as [***z***_7_–18 + H]^+•^, [***z***_7_–43]^+•^, and [***y***_7_ + 2H]^+2^, found only in the MS/MS spectrum, and [_r_***a***_4_–72 + H]^+^ and [_r_***c***_5_–99 + 2H]^+^, observed only in the MS^3^ spectrum. The assignments of the fragments appearing in both spectra indicate that ***a***-type ions were a major product ion type and that ***c***-/***z***-type ions were minor. Many neutral and radical loss peaks were also observed in the so-called (M–X) ^+•^ region below [_r_M + H]^+•^, which included 18, 29, 44, 56, 86, and 106 Da losses^24,37–40^. These neutral and radical loss peaks suggest the presence of I (–29, –56 Da), D (–44 Da), R (–86 Da), and Y (–106 Da) in the sequence^26^. These observations clearly suggest that, as expected, in the MS/MS and MS^3^ of *p*-TEMPO–Bn–Sc–DRVYIHPF conjugated peptides, radical-driven peptide backbone dissociation mechanisms governed the overall gas-phase peptide fragmentations.

More interestingly, the similarity of the MS/MS spectra and MS^3^ spectra indicates that the two consecutive processes, *i.e.*, the homolytic cleavage of the C–O bond between the benzyl carbon and the oxygen of the TEMPO group and the radical-driven peptide backbone fragmentations, consecutively occurred with only a single application of collision activation (MS/MS) of *p*-TEMPO–Bn–Sc–DRVYIHPF, in contrast to that of the *o*-TEMPO–Bz–C(O)– conjugated peptide. This result clearly demonstrates that our design rationale for the new *p*-TEMPO–Bn–Sc–peptide worked well, and different dissociation energetics occur in the case of *p*-TEMPO–Bn–Sc–DRVYIHPF. A more detailed exploration of the dissociation energetics will be discussed later.

Doubly protonated *p*-TEMPO–Bn–Sc– conjugated angiotensin II was also subjected to MS/MS and MS^3^, with the results shown in Supplementary Fig. 2a and b, respectively. Similar to singly protonated conjugated angiotensin II, doubly protonated *p*-TEMPO–Bn–Sc–angiotensin II also produced extensive peptide backbone fragments in a single-step CA experiment (see Supplementary Fig. 2a). Fragments of the ***a***-, ***c***-, ***x***- and ***z***-types are ubiquitously found in the spectrum, which indicates that the radical-driven peptide backbone fragmentation mechanism was a dominant process over the charge-driven mechanism.

### Other peptides

To further explore the characteristics of the peptide backbone fragmentations for the *p*-TEMPO–Sc–Bn–peptides, three more peptides, des-Pro^2^-bradykinin (RPGFSPFR), glycoprotein IIb fragment 296–306 (TDVNGDGRHDL), and kinetensin (IARRHPYFL), were subjected to MS/MS experiments. These peptides were used in our previous studies with the *o*-TEMPO–Bz–C(O)–NHS reagent, allowing us to compare the observed backbone dissociation behaviors of the two differently conjugated peptides (see the next subsection).

The MS/MS spectra of the singly protonated *p*-TEMPO–Bn–Sc– conjugate of (a) Pro^2^-bradykinin (RPGFSPFR), (b) glycoprotein IIb fragment 296–306 (TDVNGDGRHDL) and (c) kinetensin (IARRHPYFL) are shown in Supplementary Fig. 3. As in the angiotensin II spectrum, in all the MS/MS spectra, a large number of peptide backbone fragments were observed, particularly a series of ***a-***/***x-***type and ***c-***/***z-***type fragments with some minor ***b-***/***y-***type ions. The peptide backbone fragments found in the MS/MS spectra were also observed in the MS^3^ spectra. A few extra fragments were observed only in the MS^3^ spectra; for example, [_r_***a***_7_–90 + 2H]^+^ for Pro2-bradykinin; and [_r_***a***_8_–57 + 2H]^+^, [_r_***b***_8_–106 + 2H]^+^ and [_r_***c***_8_–106 + H]^+•^ and for glycoprotein IIb fragment.

In all three peptide spectra, the peaks arising from neutral/radical loss also appeared: arginine (–72, –86, –99/–100 Da), aspartic acid (–44 Da), isoleucine (–29, –56 Da), leucine (–43, –56 Da), serine (–30 Da), histidine (–67 Da), phenylalanine (–90 Da), and tyrosine (–92, –106 Da) side chain losses; neutral losses of NH_3_, (–17 Da), H_2_O (–18 Da), CO (–28 Da), CO_2_ (–44 Da), and •COOH + NH_3_ (–61); and neutral losses of 105, 120, and 148 Da stemming from the breakage of the relatively weak bonds of the *p*-TEMPO–Bn–Sc– radical initiator. These abundant neutral/radical losses also provide evidence that radical-driven processes are dominant in a single CA application for *p*-TEMPO–Bn–Sc–peptides. It was universally observed that a single CA was enough for *p*-TEMPO–Bn–Sc–peptides to undergo extensive radical-driven peptide backbone fragmentations.

### Comparison of *p*-TEMPO–Bn–Sc– and *o*-TEMPO–Bz–C(O)–peptide FRIPS MS

**Figure 6:**
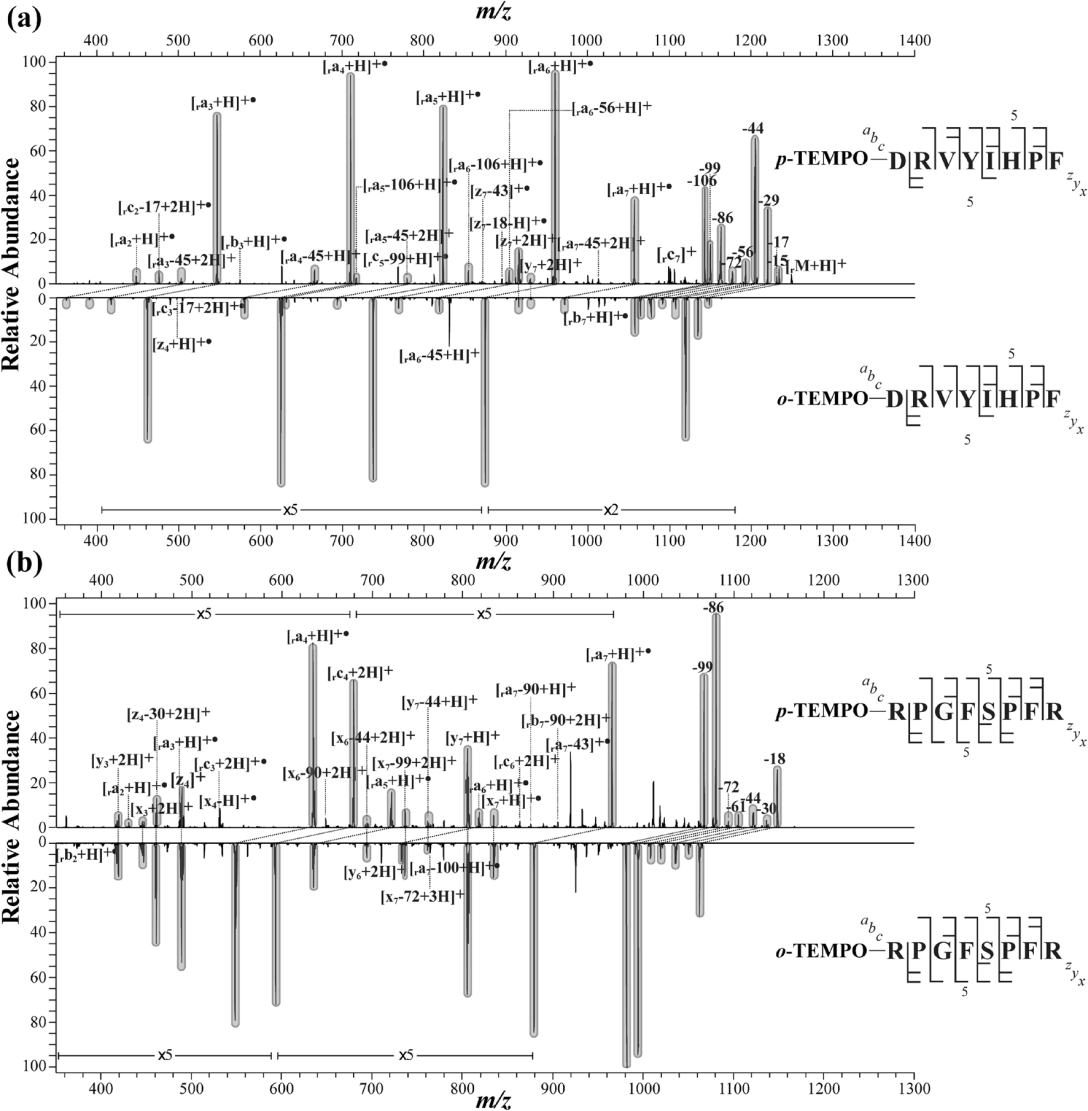
(**a**) MS^3^ spectra of *p*-TEMPO–Bn–Sc–angiotensin II (DRVYIHPF) (top) and *o*-TEMPO–Bz–C(O)–angiotensin II (bottom) and (**b**) MS^3^ spectra of *p*-TEMPO–Bn–Sc–des-Pro^2^-bradykinin (RPGFSPFR) (top) and *o*-TEMPO–Bz–C(O)–des-Pro^2^-bradykinin (bottom).

At this point, it would be informative to compare the fragmentation MS spectra of the peptides conjugated with the two different TEMPO moiety-containing reagents. As representative examples, the FRIPS MS spectra of *p*-TEMPO–Bn–Sc– and *o*-TEMPO–Bz–C(O)–angiotensin II (DRVYIHPF) and des-Pro^2^-bradykinin (RPGFSPFR) are compared in Fig. 6. Since *p*-TEMPO–Bn–Sc–NHS has a molecular mass 86 Da higher than that of *o*-TEMPO–Bz–C(O)–NHS, each N-terminal fragment, *i.e.*, _r_***a***-, _r_***b***-, or _r_***c***-ion, arising from the MS/MS of *p*-TEMPO–Bn–Sc–peptide would also be shifted by + 86 Da from the corresponding MS^3^ spectra fragment from the *o*-TEMPO–Bz–C(O)–peptide. In contrast, each C-terminal fragment would show the same *m/z* values. To facilitate the comparison, in Fig. 6, the corresponding fragment pairs with identical peptide backbone cleavages are shaded in gray.

In terms of the observed fragments and their relative abundances, the FRIPS MS fragmentation behaviors were very similar to each other for both peptides of angiotensin II and des-Pro^2^-bradykinin. The structural difference of the two radical initiators did not cause a remarkable difference in the peptide backbone fragmentations and side-chain losses, as evidenced by the gray-shaded peak-to-peak matches between the *p*-TEMPO–Bn–Sc– and the *o*-TEMPO–Bz–C(O)–peptide FRIPS MS spectra. More specifically, twenty-three and twenty-two fragment ions were commonly found in the FRIPS MS spectra of angiotensin II and des-Pro^2^-bradykinin, respectively. Only a few ions other than these common set of ions were exclusively observed in either one of the two spectra. For example, six and four fragments were found exclusively in the FRIPS MS spectrum of *p*-TEMPO–Bn–Sc– and *o*-TEMPO–Bz–C(O)–angiotensin II, respectively: for *p*-TEMPO–Bn–Sc–angiotensin II, [_r_***b***_3_ + H]^+•^, [_r_***c***_5_–99 + H]^+•^, [***z***_7_–43]^+•^, [***z***_7_–18 + H]^+•^, [_r_***a***_7_–45 + 2H]^+^ and [_r_***c***_7_]^+^; for *o*-TEMPO–Bz–C(O)–angiotensin II, [_r_***c***_3_*–*17 + 2H]^+•^, [***z***_4_ + H]^+•^, [_r_***a***_6_*–*45 + H]^+^ and [_r_***b***_7_ + H]^+^. Based on these observations, it appears that the fragmentation pathways of the *o*-TEMPO–Bz–C(O)– and *p*- TEMPO–Bn–Sc–peptide conjugates are almost identical.

It is also noteworthy that the positional isomerism of the radical initiators, *i.e.*, *o*-TEMPO–Bz–C(O)–NHS vs. *p*-TEMPO–Bn–Sc–NHS, did not greatly affect its peptide backbone fragmentation and side chain losses, as indicated in our previous study (spectra not shown).

### FRIPS MS of phosphopeptides from chicken egg albumin and alpha-casein

**Figure 7:**
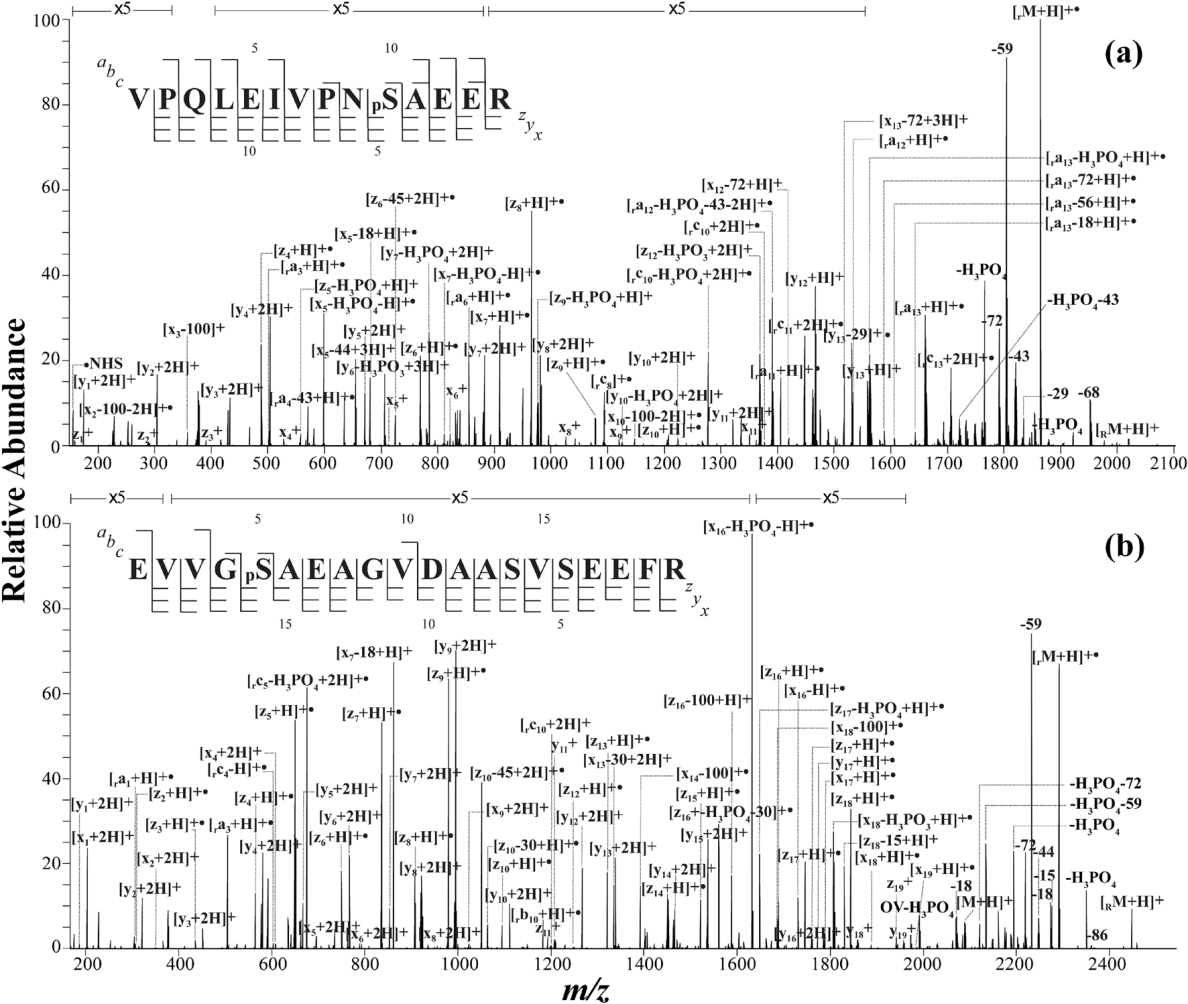
MS/MS spectrum of *p*-TEMPO–Bn–Sc– conjugated phosphopeptides from (**a**) alpha-casein (VPGLEIVPN_p_SAEER) and (**b**) chicken egg albumin (EVVG_p_SAEAGVDAASVSEEFR).

In our previous work, it was shown that the FRIPS MS of phosphopeptides conjugated with *o*-TEMPO–Bz–C(O)–NHS could achieve backbone fragmentation without the loss of a phosphate group, thus readily localizing the phosphorylated site^28^. To evaluate whether the newly developed TEMPO reagent, *p*-TEMPO–Bn–Sc–NHS, would work as *o*-TEMPO–Bz–C(O)–NHS did for the characterization of the phosphopeptides, alpha-casein and chicken egg albumin were subjected to FRIPS MS. Figure 7 shows the HCD MS/MS results for singly protonated phosphopeptides from (a) alpha-casein (VPQLEIVPN_p_SAEER) and (b) chicken egg albumin (EVVG_p_SAEAGVDAASVSEEFR) with an NCE of 25 a.u. In contrast to the MS/MS spectra of the nonconjugated phosphopeptides (Supplementary Fig. 4), in which the dephosphorylated molecular ion ([M–H_3_PO_4_]^+^) was the most abundant ion, the MS/MS spectra in Fig. 7 show neutral loss peaks (caused by dephosphorylation) to only a limited extent for both phosphopeptides*.* Both MS/MS spectra in Fig. 7 show fragment ions mostly consisting of C-terminal fragments, *i.e.*, ***x***-, ***y***-, ***z***- ions, whereas the N-terminal fragments were only found at the residues in close proximity to the free radical. The abundant presence of ***y***-ions along with radical-induced fragments (***a***-/***x***- and ***c***-/***z***- ions) is the consequence of both radical- and charge-driven fragmentations occurring simultaneously^41^. This is consistent with the results from other studies where charge-driven fragments have been observed from the fragmentation of free radical initiators conjugated to peptides^28,41–45^. It is noteworthy that the ion pairs indicating the loss and the retainment of a phosphate group in both phosphopeptides, *i.e.*, [***x***_7_ + H]^+•^/[***x***_7_–H_3_PO_4_–H]^+•^, [_r_***c***_10_ + 2H]^+•^/[_r_***c***_10_–H_3_PO_4_ + 2H]^+•^, and [***z***_17_ + H]^+•^/[***z***_17_–H_3_PO_4_ + H]^+•^, allow the precise localization of the phosphate groups along the sequence.

It is also interesting to note that the dephosphorylation behavior in the FRIPS MS changes a little as the charge state increases. Specifically, the MS/MS spectra of doubly protonated *p*-TEMPO–Bn–Sc– conjugated phosphopeptides (Supplementary Fig. 5) show an increase in the number of radical-driven fragments with the loss of a phosphate group, compared with those of singly protonated ones. However, the abundances of the fragment ions with the loss of a phosphate group did not increase significantly with its charge. Further, it is noticeable in the MS/MS spectrum of the doubly protonated species that most of the ion pairs with and without the phosphate group came together from the ***y***-type ions, *i.e*., [***y***_7_ + 2H]^+^/[***y***_7_–H_3_PO_4_ + 2H] + , [***y***_11_ + H]^+^/[***y***_11_–H_3_PO_4_ + 2H]^+^, [***y***_13_ + H]^+^/[***y***_13_–H_3_PO_4_ + 2H]^+^, [***y***_16_ + 2H]^+^/[***y***_16_–H_3_PO_4_ + 2H]^+^, [***y***_17_ + 2H]^+^/[***y***_17_–H_3_PO_4_ + 2H]^+^.

For the comparison with ETD MS, EThcD was carried out for the triply protonated phosphopeptides (Supplementary Fig. 6). As expected, the number of the fragment ions with the phosphate group loss was very limited in the EThcD MS/MS spectra^46^. In terms of the number of backbone fragments, which is crucial in sequencing peptides, a comparable number of radical-driven fragments were observed in the FRIPS MS and EThcD MS for phosphopeptides. However, ***x***-type ions, which were very abundant in the FRIPS MS spectra, were rarely observed in the EThcD spectra. Another interesting difference lies in that in EThcD MS, the loss of a phosphate group was accompanied with the peptide backbone dissociation at the phosphoserine (_p_S) residue, *i.e*., [***a***_5_–H_3_PO_4_ + H]^+^, [***a***_10_–H_3_PO_4_ + H]^+•^, and [***z***_16_–H_3_PO_4_ + H]^+^, while, in the FRIPS MS spectra (Fig. 7), the phosphate group loss was observed for the fragment ions, in which the backbone cleavage occurred at least one residue away from phosphoserine, *i.e.*, [***y***_6_–H_3_PO_4_ + 2H]^+^, [***y***_7_–H_3_PO_4_ + 2H]^+^, [_r_***a***_13_–H_3_PO_4_ + H]^+•^, [***z***_17_–H_3_PO_4_ + H]^+•^ and [***x***_18_–H_3_PO_4_ + H]^+•^. In addition, as the application of ETD involves the charge reduction, no and only a few fragment ions were observed in the EThcD MS/MS spectra for singly and doubly protonated phosphopeptides, respectively, which is in stark contrast to the FRIPS MS results.

To summarize, with the improved conjugation efficiency and the single-step FRIPS MS implementation, the FRIPS MS approach with *p*-TEMPO–Bn–Sc– conjugation could expand its applicability in the proteomic workflow of phosphopeptide analysis.

## Conclusion

FRIPS MS was successfully implemented using a newly designed *p*-TEMPO–Bn–Sc– radical initiator, particularly with only a single application of MS/MS, even in positive ion mode. The survival fraction analysis as a function of the applied NCE showed a reversed energy ordering, in which the NCE_1/2_ required in the first CA was higher than that in the second CA. This reversal is likely to be the main thermodynamic driving force leading to extensive radical-driven peptide backbone dissociations at the single-step CA. Consistent with previous *o*-TEMPO–Bz–assisted FRIPS MS studies, the FRIPS MS of the *p-TEMPO*–*Bn*–Sc–peptides also resulted in extensive radical-driven peptide backbone dissociations. Collisional activation of *p-TEMPO*–*Bn*–Sc–peptides led to the generation of *p*-TEMPO• radicals and •Bn–Sc–peptides by C–O homolytic cleavage and to subsequent peptide backbone dissociations, yielding a large number of ***a***-, ***x***-, ***c***-, and ***z***-type fragment ions and small neutral losses. As a result, the new TEMPO MS approach using the *p-TEMPO*–*Bn*–Sc– radical initiator can improve the duty cycle in positive-ion mode. We have also shown that the new FRIPS MS approach using *p-TEMPO*–*Bn*–Sc–NHS for phosphopeptides preserves phosphate groups, thus allowing for precise localization of the phosphate groups along the peptide sequence. This clearly demonstrates the potential to expand the applicability of FRIPS MS to the proteomic workflow of phosphopeptide analysis.

## Supplementary Information

Supplementary Figure 1.

## Data Availability

The mass spectrometric datasets generated and/or analysed during the current study are available in the figshare repository, https://figshare.com/s/511b79fc1857c682f1e6. Also, the protein sequences for two phosphoproteins are available in the uniport repository (http://uniprot.org) with following uniprot protein entry number, P02663 (bovine alpha casein) and P01012 (chicken egg albumin).
