# Author Correction: New free radical-initiated peptide sequencing (FRIPS) mass spectrometry reagent with high conjugation efficiency enabling single-step peptide sequencing

**DOI:** 10.1038/s41598-023-32194-3

**Published:** 2023-03-28

**Authors:** Sang Tak Lee, Hyemi Park, Inae Jang, Choong Sik Lee, Bongjin Moon, Han Bin Oh

**Affiliations:** 1 Department of Chemistry, Sogang University, Seoul, 04107 Korea; 2 Department of Toxicology and Chemistry, Scientific Investigation Laboratory, Criminal Investigation Command, Ministry of National Defense, Seoul, 04351 Korea

Correction to: *Scientific Reports* 10.1038/s41598-022-13624-0, published online 09 June 2022

The original version of this Article contained an error in the Acknowledgments section.

"This work is supported by the National Research Foundation of Korea (NRF) grant funded by the Korea government (MSIT) (2021R1A2C2007397 and 2017M3D9A1073784), the Ministry of Education (2018R1A6A1A03024940) through Basic Science Research Program and the Korea Ministry of Environment (MOE) (2020002960004). We are also thankful for the financial support by a grant of the Korea Health Technology R&D project through the Korea Health Industry Development Institute (KHIDI), funded by the Ministry of Health & Welfare, Republic of Korea (Grant Number HI17C1238030021)."

now reads:

"This work is supported by the National Research Foundation of Korea (NRF) grant funded by the Korea government (MSIT) (2021R1A2C2007397 and 2017M3D9A1073784), the Ministry of Education (2018R1A6A1A03024940) through Basic Science Research Program and the Korea Ministry of Environment (MOE) (2020002960004). We are also thankful for the financial support by a grant of the Korea Health Technology R&D project through the Korea Health Industry Development Institute (KHIDI), funded by the Ministry of Health & Welfare, Republic of Korea (Grant Number HI17C1238)."

The original Article has been corrected.

