# Supplementary material for: New free radical-initiated peptide sequencing (FRIPS) mass spectrometry reagent with high conjugation efficiency enabling single-step peptide sequencing
Source: Sci Rep. 2022 Jun 9;12:9494. doi: 10.1038/s41598-022-13624-0 (PMC9184593; doi:10.1038/s41598-022-13624-0)
Supplement: Supplementary file 1 — Supplementary Figure 1. [file 41598_2022_13624_MOESM1_ESM.pdf]

Supporting Information

New Free Radical-Initiated Peptide Sequencing (FRIPS) Mass Spectrometry Reagent with High Conjugation Efficiency Enabling Single-Step Peptide Sequencing

Sang Tak Lee[1], Hyemi Park[1], Inae Jang[1], Choong Sik Lee[1,2], Bongjin Moon[1*], and Han Bin Oh[1*]

[1]Department of Chemistry, Sogang University, Seoul 04107, Korea

[2]Department of Toxicology and Chemistry, Scientific Investigation Laboratory, Criminal Investigation Command, Ministry of National Defense, Seoul 04351, Korea

KEYWORDS: free radical-initiated peptide sequencing (FRIPS), TEMPO, succinic acid, radical ion, mass spectrometry

[#]Both authors equally contributed to this work.

Corresponding Authors

*To whom correspondence should be addressed. E-mail: hanbinoh@sogang.ac.kr (Prof. Han Bin Oh) and bjmoon@sogang.ac.kr (Prof. Bongjin Moon).

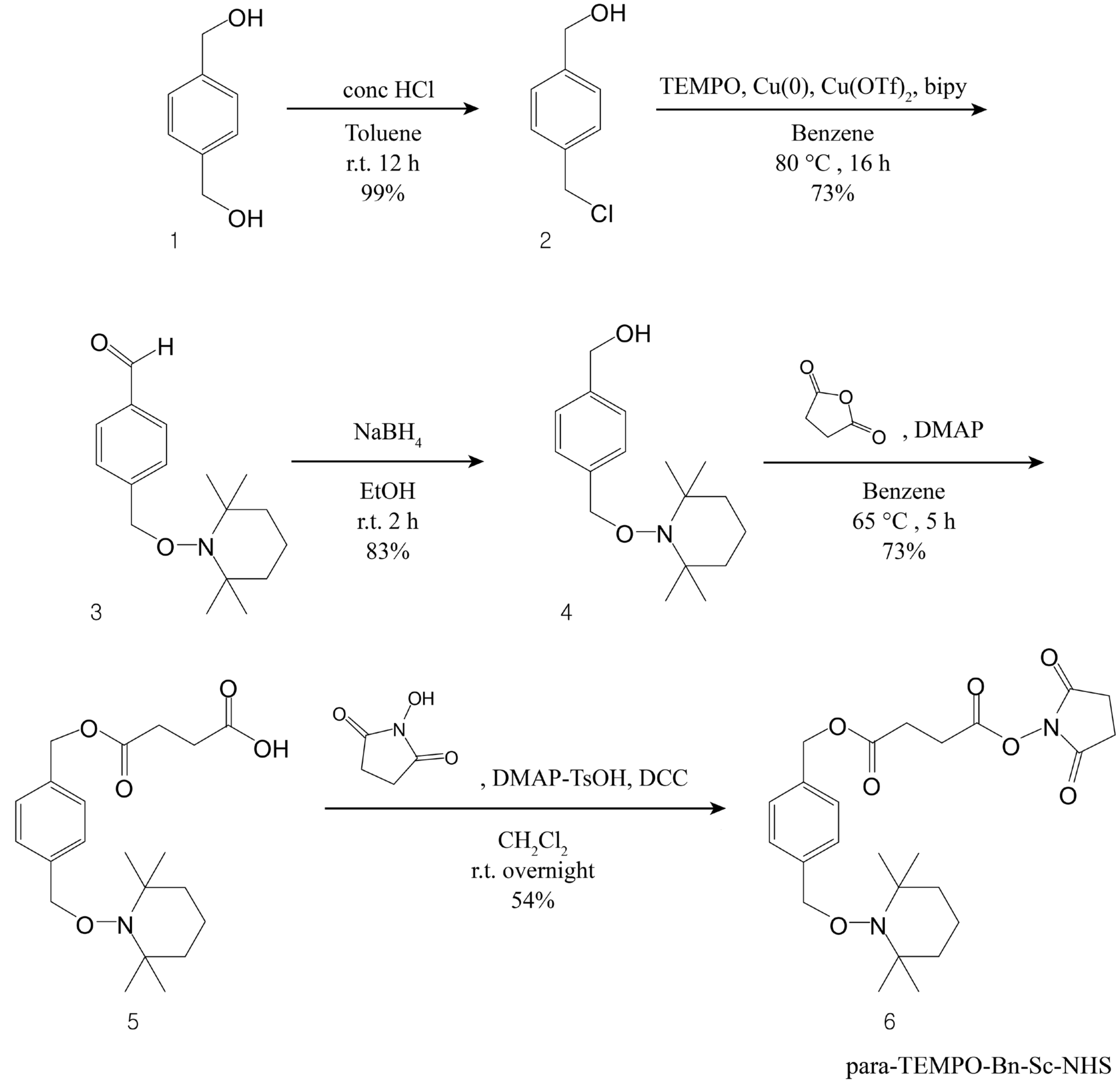

Figure S1. Synthesis of *p*-TEMPO–Bn–Sc–NHS

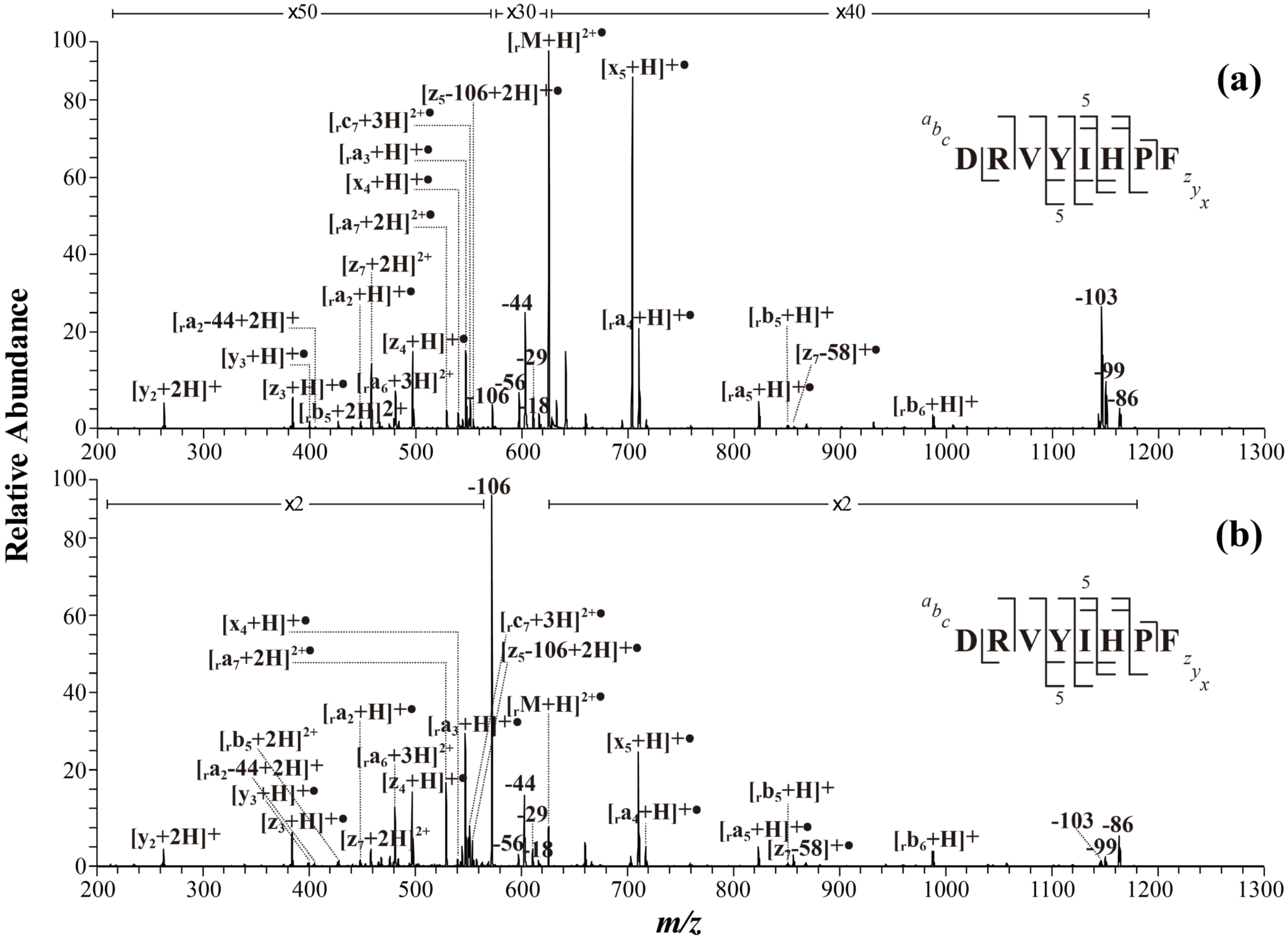

Figure S2. (a) MS/MS and (b) MS^3 spectra of doubly protonated *p*-TEMPO–Bn–Sc–angiotensin II (DRVYIHPF) at normalized collision energy of 25 (a.u.).

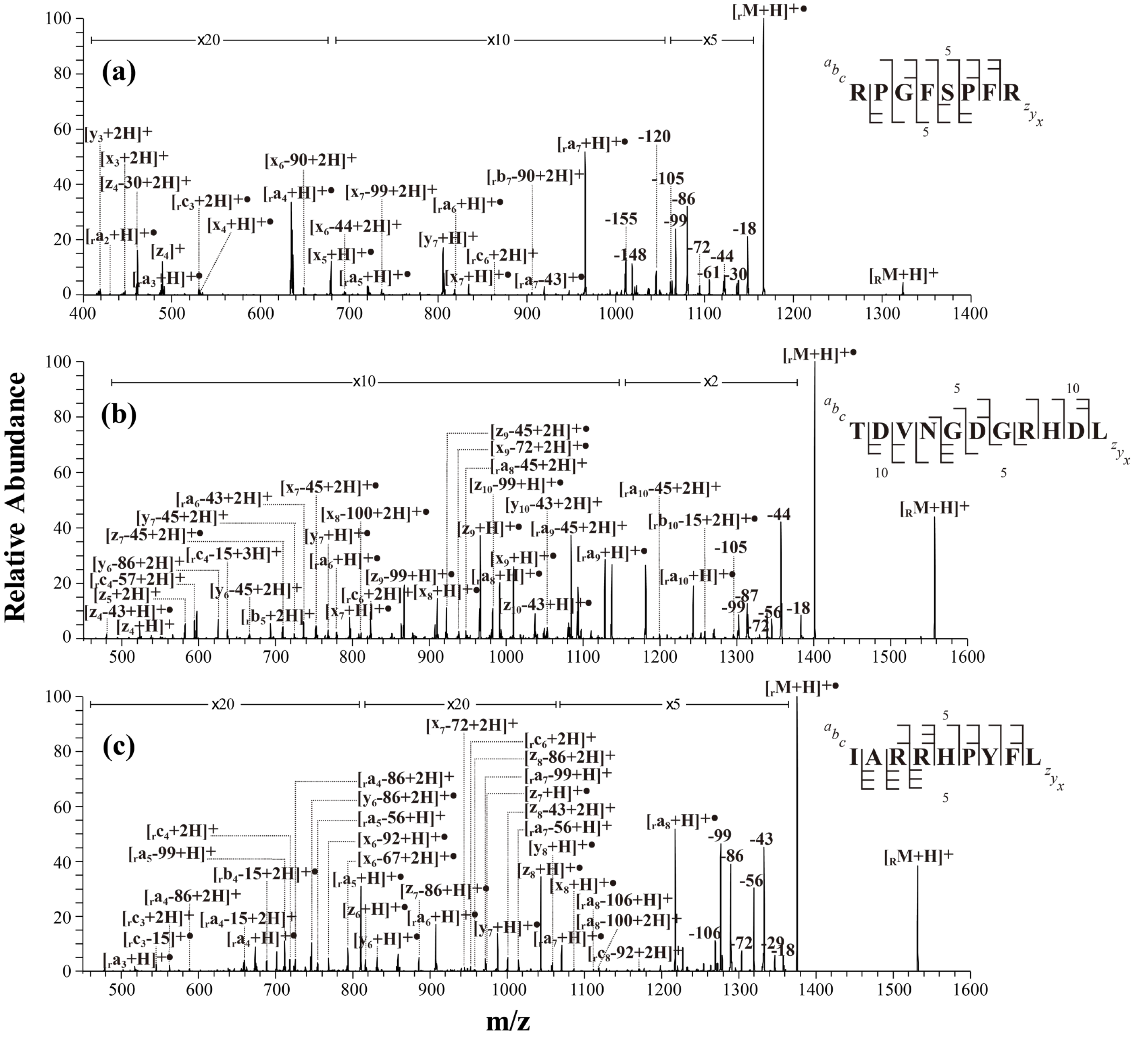

Figure S3. MS/MS spectra of singly protonated (a) *p*-TMEPO–Bn–Sc–des-Pro^{2}-bradykinin (RPGFSPFR), (b) *p*-TEMPO–Bn–Sc–glycoprotein IIb fragment 296-306 (TDVNGDGRHDL) and (c) *p*-TEMPO–Bn–Sc–kinetensin (IARRHPYFL) at normalized collision energy of 25 (a.u.).

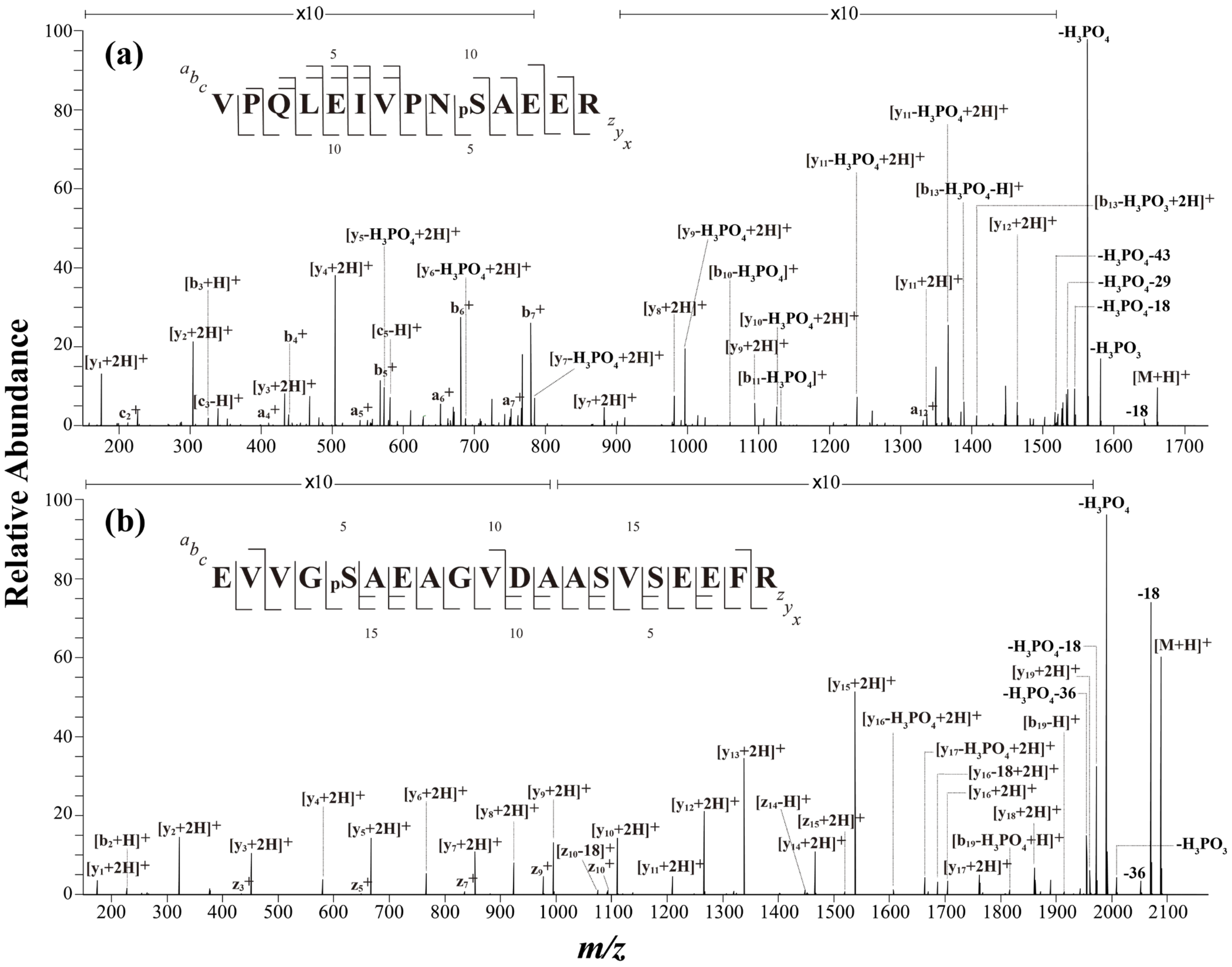

Figure S4. MS/MS spectrum of singly protonated phosphopeptides from (a) alpha-casein (VPGLEIVPNS$_p$AEER) and (b) chicken egg albumin (EVVGS$_p$AEAGVDAASVSEEFR)

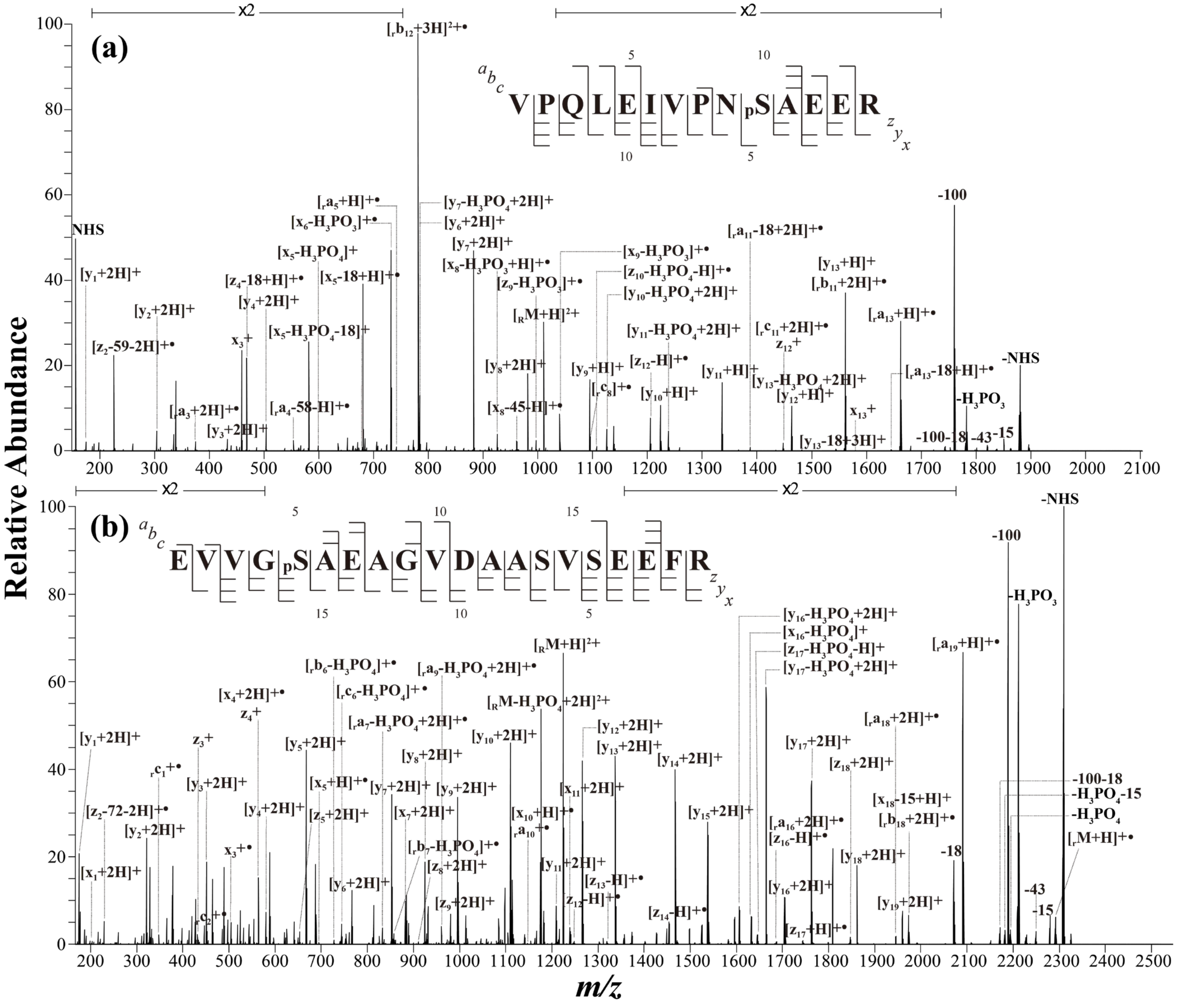

Figure S5. MS/MS spectra of doubly protonated *p*-TEMPO–Bn–Sc– conjugated phosphopeptides from (a) alpha-casein (VPGLEIVPN$_p$SAEER) and (b) chicken egg albumin (EVVG$_p$SAEAGVDAASVSEEFR)

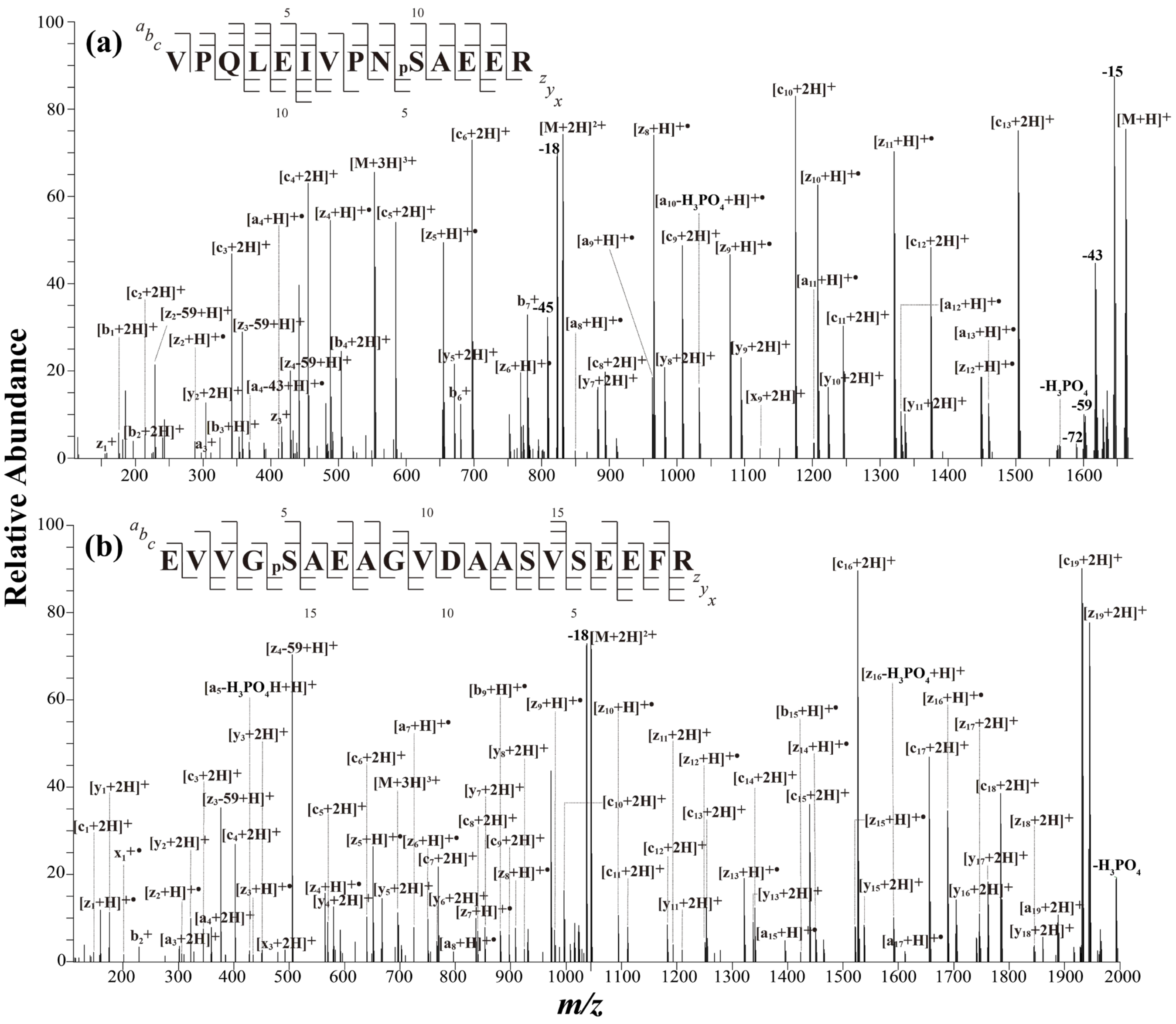

Figure S6. Electron-transfer/higher-energy collision dissociation (EThcD) MS/MS spectra of triply protonated *p*-TEMPO–Bn–Sc– conjugated phosphopeptides from (a) alpha-casein (VPGLEIVPN$_{p}$SAEER) and (b) chicken egg albumin (EVVG$_{p}$SAEAGVDAASVSEEFR)
